# Stair negotiation made easier using novel interactive energy-recycling assistive stairs

**DOI:** 10.1371/journal.pone.0179637

**Published:** 2017-07-12

**Authors:** Yun Seong Song, Sehoon Ha, Hsiang Hsu, Lena H. Ting, C. Karen Liu

**Affiliations:** 1 Department of Mechanical Engineering, Missouri University of Science and Technology, Rolla, Missouri, United States of America; 2 Disney Research, Pittsburgh, Pennsylvania, United States of America; 3 Department of Mechanical Engineering, Georgia Institute of Technology, Atlanta, Georgia, United States of America; 4 Department of Biomedical Engineering, Georgia Institute of Technology and Emory University, Atlanta, Georgia, United States of America; 5 Department of Rehabilitation Medicine, Division of Physical Therapy, Emory University, Atlanta, Georgia, United States of America; 6 School of Interactive Computing, Georgia Institute of Technology, Atlanta, Georgia, United States of America; Fondazione Santa Lucia Istituto di Ricovero e Cura a Carattere Scientifico, ITALY

## Abstract

Here we show that novel, energy-recycling stairs reduce the amount of work required for humans to both ascend and descend stairs. Our low-power, interactive, and modular steps can be placed on existing staircases, storing energy during stair descent and returning that energy to the user during stair ascent. Energy is recycled through event-triggered latching and unlatching of passive springs without the use of powered actuators. When ascending the energy-recycling stairs, naive users generated 17.4 ± 6.9% less positive work with their leading legs compared to conventional stairs, with the knee joint positive work reduced by 37.7 ± 10.5%. Users also generated 21.9 ± 17.8% less negative work with their trailing legs during stair descent, with ankle joint negative work reduced by 26.0 ± 15.9%. Our low-power energy-recycling stairs have the potential to assist people with mobility impairments during stair negotiation on existing staircases.

## Introduction

Stair negotiation is a demanding task that limits the independence of individuals with mobility impairments such as muscle weakness, joint pain, or reduced sensorimotor control. Joint moments in the knee are over 3 times greater during stair negotiation compared to level walking during both stair ascent and descent [1–3]. Stair negotiation is ranked among the top 5 most difficult tasks in community-residing older adults [4, 5]. Patients—such as those with hip osteoarthritis—adopt altered joint movements to reduce pain during stair negotiation [6]. Moreover, even if they are capable of using stairs, people with mobility impairments often avoid stair negotiation [5, 7].

Current solutions providing assistance in stair negotiation are costly, energy-consuming, and do not help to retain the user’s ability to negotiate stairs independently. Elevators or stair-lifts are often impractical to install because they require substantial household remodeling. Further, an elevator can consume over 12,000 kWh annually [8], equivalent to 50% of the average household energy consumption in the United States in 2009 [9] and over 200% of that in the United Kingdom in 2004 [10]. Perhaps more importantly, elevators or stair-lifts replace the need to negotiate stairs altogether, regardless of a user’s level of motor function. Because studies suggest that disuse of a specific motor function can further accelerate its loss [11–13], it is important to provide motor assistance that allows users to retain their ability to use stairs and to prevent further motor decline.

**Fig 1:**
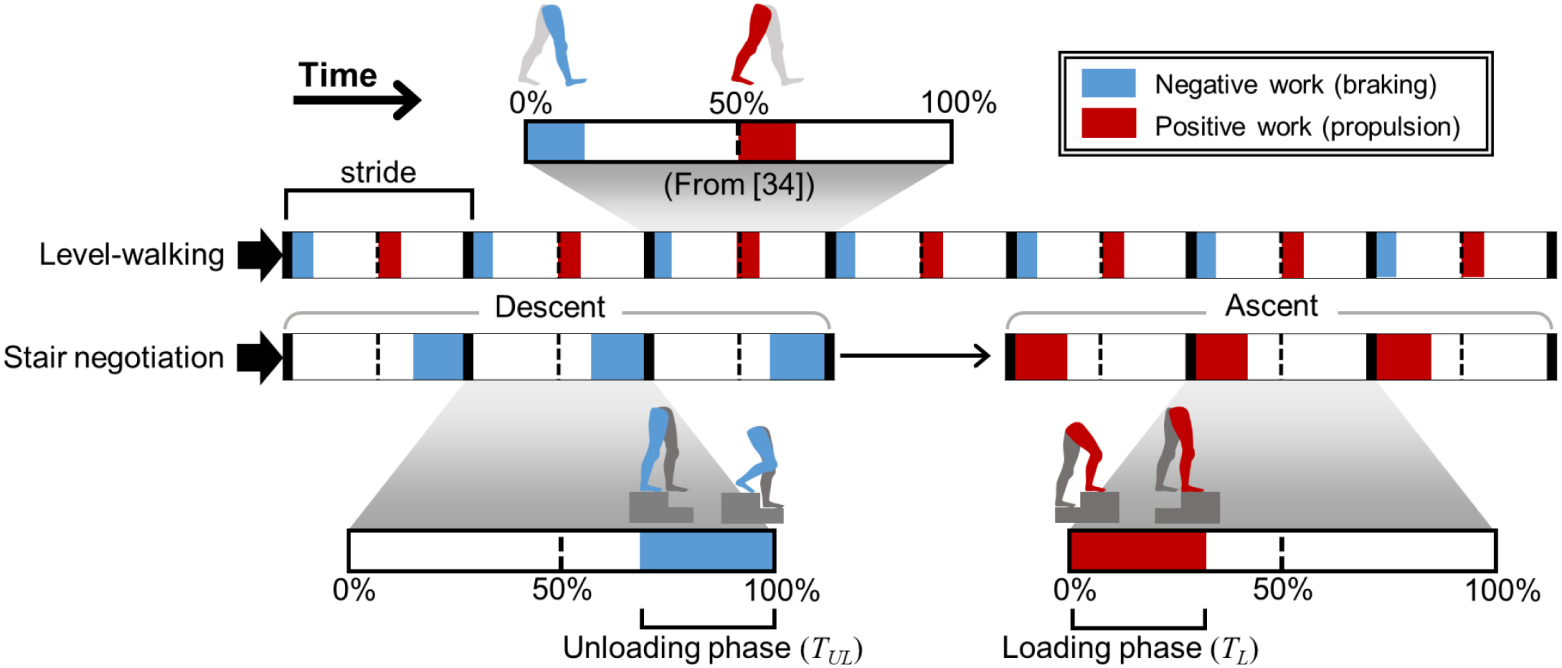
Positive and negative work generation during walking and during stair negotiation. Blue represents the phases during which negative work is generated by the right leg (braking). Red represents the phases during which positive work is generated by the right leg (propulsion). In walking, the right leg both brakes and propels within one stride. In stair negotiation, however, the right leg generates predominantly negative work throughout stair descent (*T*_*UL*_), and predominantly positive work throughout stair ascent (*T*_*L*_). The gait cycle during stair negotiation follows the definition in [1].

Cheap, low-power, yet effective human movement assistance is possible by applying the principle of energy recycling [14]. Collins et al. showed that a simple exoskeleton with passive springs can store and return energy at each step to assist joint motion during walking [15]. The exoskeleton takes advantage of the alternating braking and propelling action of the legs during gait at the level of the ankle (Fig 1). During braking, the lower-leg exoskeleton stores mechanical energy in a passive spring, reducing the negative work generated by the ankle to brake the leg. The stored energy is later released to propel the body forward, reducing the positive work generated by the ankle for propulsion. Through appropriate detection and response to gait events, the exoskeleton reduces the metabolic cost of walking by 7.2% without consuming energy.

In contrast to level walking, storing and returning energy within each gait cycle cannot be applied to stair negotiation. The legs produce predominantly positive work during stair ascent, and predominantly negative work during stair descent [2]. Thus, a more effective approach for energy-recycling in stair negotiation would be to store a large amount of energy cumulatively during stair descent, and to then release that energy during stair ascent. Specifically, energy recycling could be targeted to two phases where the greatest increase in joint power are observed compared to level walking: the loading phase of stair ascent (*T*_*L*_) when energy is *generated* by the leading leg, and the unloading phase of stair descent (*T*_*UL*_) when energy is *absorbed* by the trailing leg (Fig 1). In the loading phase (*T*_*L*_), center-of-mass (CoM) elevation occurs early in the gait cycle, between foot strike of the leading leg and into the mid swing phase of the trailing leg. Sagittal knee joint power throughout *T*_*L*_ is higher than in level walking, both in adults over 40 years old [3] and healthy young adults [2], with peak knee power of roughly 2 times greater than in level walking [1, 2]. In the unloading phase (*T*_*UL*_), the center-of-mass (CoM) is lowered as the trailing leg does negative work (absorbs energy) until toe-off. In healthy young adults, knee joint power throughout *T*_*UL*_ is higher than in level walking [2], and peak knee joint power reaches 3.8 times that seen during level walking [1, 2]. Further, the ankle joint generates large negative power in sagittal plane during *T*_*UL*_ but negligible negative power during overground walking. Based on previous findings [1–3], we hypothesized that assistive stairs could store energy during *T*_*UL*_ and release it during *T*_*L*_, reducing both positive (ascent) and negative (descent) work generation in the legs during stair negotiation.

Therefore, our objective was to design, build, and test energy-recycling assistive stairs (ERAS) that store energy during stair descent and return it to assist the user during stair ascent. We built two prototype modular steps that can be placed on an existing staircase. Energy is stored in passive springs and released based on gait events detected by pressure sensors on each tread. We then measured the amount of work generated or dissipated by the leg joints during assisted and unassisted stair negotiation in naive users. Our results show that ERAS reduces positive work generation in the leading leg during ascent by 17.4 ± 6.9%. In particular, a 37.7 ± 10.5% reduction was observed in the knee, which is one of the main contributors of positive work during ascent. In addition, ERAS reduces negative work generation in the trailing leg during descent by 21.9 ± 17.8%. In particular, a 26.0 ± 15.9% reduction was observed in the ankle-sagittal degrees-of-freedom (DOF), which is one of the main contributors of negative work during descent. Together, our work demonstrates the feasibility of a low power, modular, interactive device to assist those with difficulty in stair negotiation in their homes.

## Materials and methods

### Energy-recycling assistive stairs

**Fig 2:**
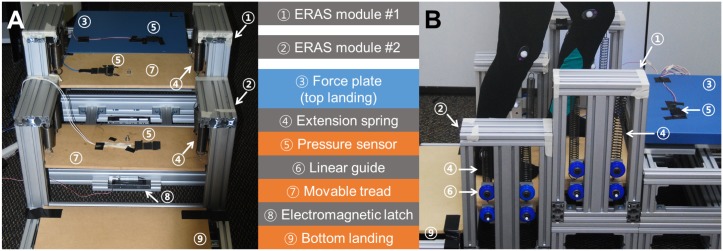
Two ERAS modules with the top and bottom landings. A) Front view, B) Side view with a user ascending the ERAS. Each ERAS module consists of its own set of extension springs, a pressure sensor, linear guide, movable tread and an electromagnetic latch. The two modules are operated by a single Arduino board (not shown). A force plate at the top landing measures the ground reaction forces of a human user.

Each ERAS module is equipped with its own latch, sensor, and a set of springs (Fig 2). Each is a single stair step designed to be placed on an existing stair with step height (17 cm) and depth (28 cm). Customizable aluminum frames (80/20^®^ Inc.), encase a movable tread, linear guides, four extension springs, an electromagnetic latch, and a pressure sensor. The tread motion is constrained to be vertical by linear guides with roller bearings (80/20^®^ Inc.). Four extension springs (Century Springs Corp.) connect the movable tread to the frame and are extended as the tread is lowered. ERAS provides three choices of stiffness for each individual spring: 350 N/m, 560 N/m, and 910 N/m. The highest total stiffness is 3640 N/m, which allows users up to 122 kg to use the current ERAS prototype. When the tread is fully lowered, it contacts an electromagnetic latch at the bottom (Docooler H10054, 180 kg holding force). Pressure sensors (Interlink Electronics^®^) detect foot placement during both ascent and descent. See S1 Video for ERAS operation.

Based on user feedback in pilot studies, we set the effective spring constant of each ERAS tread to be *k*_*norm*_ = 30.80 ± 1.3 N/m per kg of body weight. Using this weight-dependent spring stiffness, *η* = 26.7 ± 1.1% of the potential energy lost while descending a step height of *h* = 17 cm (Δ*E*_*potential*_ [J/kg]), is stored in the extended spring (Δ*E*_*spring*_ [J/kg]), such that $\Delta E_{spring}=\frac{1}{2}k_{norm}h^{2}=\eta gh=\eta \Delta E_{potential}$. Note that when the springs are removed or their motion is locked, ERAS modules do not recycle energy (Δ*E*_*spring*_ = 0) and are therefore equivalent to a normal set of stairs.

**Fig 3:**
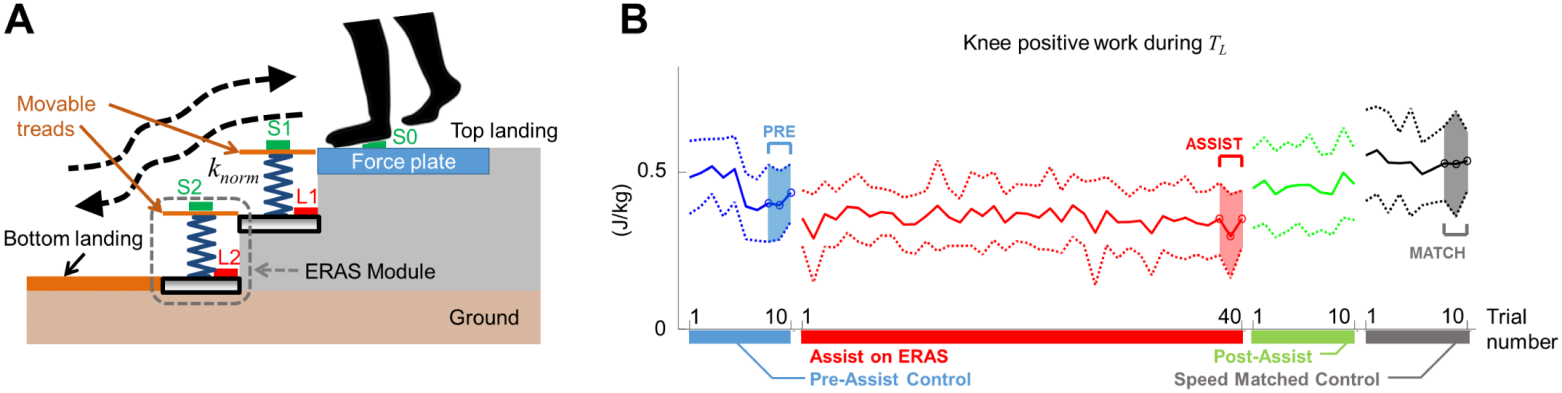
Overview of ERAS human user experiment. A) Schematic of the ERAS setup. The pictured compression springs are physically implemented in hardware using extension springs (see Fig 2). Participants start each trial on the top landing, storing energy in the springs as they descend the steps. Energy stored in the springs is released back to the user as they ascend the steps. L1 and L2 are the electromagnetic latches, whereas S0, S1 and S2 are the pressure sensors. B) Positive work generated by the knee over *T*_*L*_ in each trial over an entire experimental session. Each experiment consisted of 10 pre-assist control trials (blue), 40 assist trials (red), 10 post-assist trials (green) and 10 speed-matched control trials (gray). Solid lines denote mean and thin lines denote one standard deviation across all participants.

We prepared a staircase for human experiments with a top landing with a force plate, two ERAS, and bottom landing (Figs 2 and 3). The first (top) ERAS module was elevated 17 cm above the ground, while the second (bottom) ERAS module was directly on the ground. The 120-cm-long bottom landing was 9.2 cm above the ground to match the lowest position of the movable tread on the second ERAS. The 170-cm-long top landing was 43.2 cm above the ground to match the highest position of the movable tread of the first ERAS. A force plate (AccuGait^®^, Advanced Mechanical Technology, Inc.) formed part of the top landing directly adjacent to the first ERAS. This allowed the measurement of ground reaction forces (GRFs) on the non-mobile top landing instead of on the movable tread, on which the force readings would be affected by and interfere with tread movement. Note that our measures of joint work were restricted to the loading or unloading phases of the gait cycle in the initial (descent) or final (ascent) steps.

The ERAS was designed to be interactive with the user. The electromagnetic latches of the ERAS are modulated based on the inputs from the pressure sensor using a simple binary controller on a single Arduino^®^ Uno board. Prior to use (home-state), both latches on the first and the second ERAS (L1 and L2, respectively, Fig 3) are off, with no load detected by the pressure sensors on the force plate, the first ERAS, nor the second ERAS (S0, S1, and S2, respectively, Fig 3). During stair descent, a user steps on S1 which triggers L1 to turn on, locking the movable tread in the lowered position. On the next descending step, the user steps on S2 which then triggers L2 to turn on, latching the next movable tread in the lowered position. During a subsequent stair ascent, stepping on S2 does not trigger an event. On the next ascending step, S1 is pressed which turns L2 off, releasing the movable tread on the second (lower) ERAS. On the next ascending step, S0 is pressed which turns L1 off, releasing the movable tread on the first ERAS. Hence after a stair descent followed by an ascent, the two ERAS are returned back to their home-state and ready for the next descent to occur.

The ERAS was also designed to be modular, low-cost and energy-efficient. ERAS modules can be customized in size and shape and installed on top of existing staircases. The nonstructural components of a single ERAS unit, i.e. sensor, latch, and springs, cost less than $50 and consumes less than 5 W of electricity when a latch is on, and no external power when the latch is off.

### Human experiment

We recruited healthy young participants with no prior knowledge of the ERAS (*n* = 9, 81.0 ± 4.5 kg, 31.1 ± 4.5 yrs old, 1 female, Table 1). All participants provided their written consent to the experiment protocol, which was approved by the Institutional Review Board of Georgia Institute of Technology. All methods, including the experiments, were performed in accordance with the relevant guidelines and regulations of the board.

**Table 1:** Participants’ gender, age, weight, total spring constant, and the weight-normalized spring constant of the ERAS used in the experiments.

Participant	Gender	Age	Weight	Total spring constant	Normalized spring constant
		(yrs)	(kg)	(N/m)	*k*_*norm*_ (N/m/kg)
1	M	30	79	2521.83	31.92
2	M	27	100	2942.13	29.42
3	F	37	46	1401.01	30.46
4	M	25	94	2942.13	31.30
5	M	31	83	2521.83	30.38
6	M	35	85	2521.83	29.67
7	M	33	86	2521.83	29.32
8	M	36	80	2521.83	31.52
9	M	26	76	2521.83	33.18
Mean ± STD	N/A	31.1 ± 4.5	81.0 ± 15.1	N/A	30.80 ± 1.3

To measure how participants negotiated stairs without assistance, they first used the ERAS with the treads immobilized, i.e. energy-recycling turned off, for 10 pre-assist control trials (Fig 3). Next, the springs were connected to the treads which were allowed to move, thereby allowing the springs to store and release energy. Participants were allowed two trials (not analyzed) to familiarize themselves with the operation of the ERAS. Following this, each participant experienced 40 assist trials (30 trials for participant 6) with the springs adjusted to their body weight (*k*_*norm*_, see Table 1). The springs were then removed in 10 post-assist trials to wash out after-effects (if any) from using the ERAS before the next trials began. To ensure comparison across similar stair negotiation speeds when using ERAS [16–18], participants performed 10 additional trials with the springs removed in which they matched their step duration of the last assist trial (speed-matched control trials). The beats-per-minute (BPM) of the step cadence in the last assist trial was provided to the participant by and audio beat for approximately 1 minute after the post-assist trials. The audio was turned off prior to the speed-matched control trials to prevent participants from marching to the beat. In all other trials, participants self-selected their gait speeds. To avoid averaging transient behaviors over multiple trials during analysis, we selected only the last three pre-assist control trials (PRE), the last three assist trials (ASSIST), as well as the last three speed-matched control trials (MATCH) for analysis. The blocked conditions were designed to account for the possibility that subject would adapt their stair negotiation strategy on ERAS over repeated exposure, a common phenomenon observed when humans are exposed to novel environmental effects [19–21].

To measure GRFs on the top landing and body segment kinematics, we used a six-axis force plate (AccuGait^®^, Advanced Mechanical Technologies, Inc.) synchronized with full-body kinematics using a motion-capture system (Vicon^®^). The force plate provided the GRF and the center-of-pressure (CoP) at 120 Hz, whereas the motion-capture system provided the subject’s full-body kinematics in a motion-capture suit (53 markers), the location of the force plate, and the location of the ERAS tread, also at 120 Hz.

#### Gait phases definition

**Fig 4:**
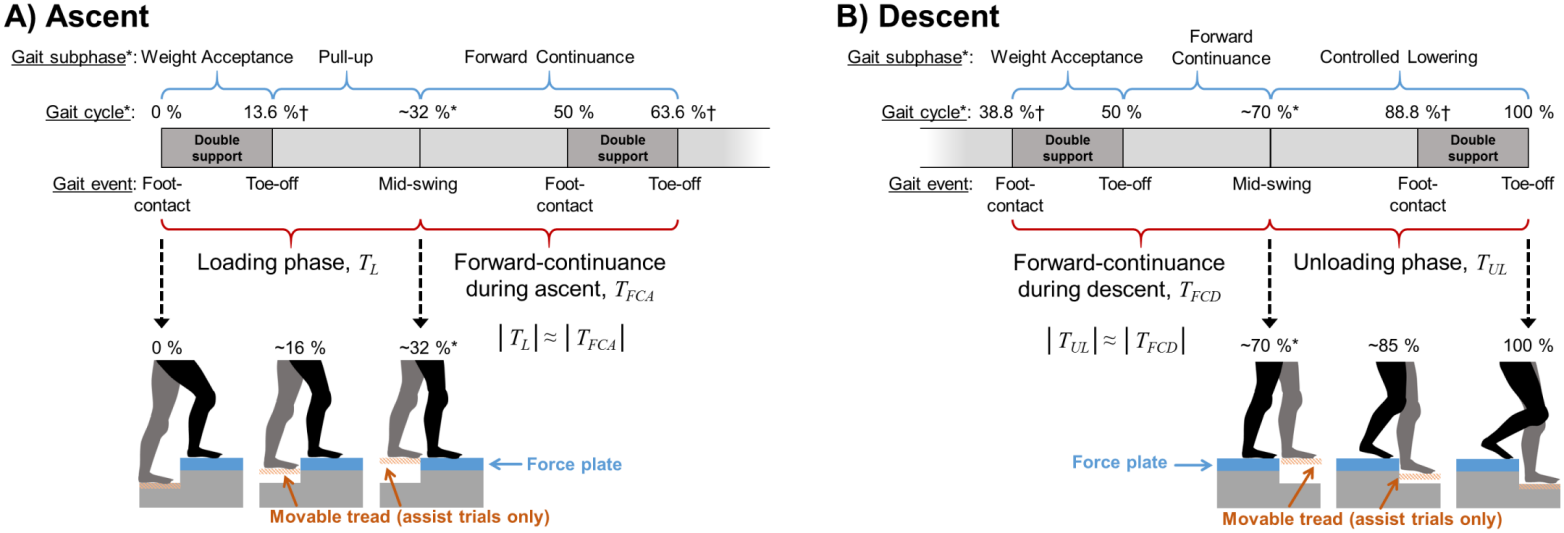
Gait phases during stair negotiation. A) During ascent, *T*_*L*_ begins with the leading foot contact (0%) and ends at mid-swing of the trailing leg (∼32%*). The forward-continuance phase, *T*_*FCA*_, begins at mid-swing and ends at the end of the double-support phase (63.6%†). B) During descent, *T*_*UL*_ begins at the second mid-swing of the leading leg (∼70%*) and ends at the trailing leg toe-off (100%). The forward-continuance phase, *T*_*FCD*_, begins with the foot contact (38.8%†) and ends at the mid-swing. *: as defined in [1]. †: as defined in [2].

We measured joint work for ascent during *T*_*L*_ and that for descent during *T*_*UL*_. The definitions of *T*_*L*_ and *T*_*UL*_ follow the gait cycle segmentation in [1] (see Fig 4). During ascent, *T*_*L*_ was identified as the time interval that began with the leading foot contact on the force plate (0% of the gait cycle) and ended as the trailing foot was lifted to the height of the leading foot (∼32%), equivalent to the combination of “Weight Acceptance” and “Pull Up” phases in [1]. During descent, *T*_*UL*_ was identified as the time interval that began at the second mid-swing of a gait cycle (∼70%) and ended with the trailing foot toe-off (100%), equivalent to the “Controlled Lowering” phase defined in [1]. Measurements from the force plate and the motion-capture system were used to identify *T*_*L*_ and *T*_*UL*_.

To test whether using ERAS affected stair negotiation outside of *T*_*L*_ or *T*_*UL*_, we defined the time interval *T*_*FCA*_ (where *FC* stands for forward-continuance [1] and *A* stands for ascent) and *T*_*FCD*_ (where *D* stands for descent, Fig 4). *T*_*FCA*_ began at the end of *T*_*L*_ (at ∼32% [1]) and ended at the end of the subsequence double-stance (63.6% [2]), making |*T*_*FCA*_| ≈ |*T*_*L*_|. Note that *T*_*FCA*_ is equivalent to the “Forward Continuance” phase during ascent defined in [1]. *T*_*FCD*_ began at the foot contact (38.8% [2]) and ended at the beginning of *T*_*UL*_ (∼70% [1]) making |*T*_*FCD*_| ≈ |*T*_*UL*_|. Note that *T*_*FCD*_ is equivalent to the combination of “Weight Acceptance” and “Forward Continuance” phases during decent defined in [1]. During *T*_*FCA*_ and *T*_*FCD*_, the treads on the ERAS were not moving since there were no feet placed on the treads. With our measurement, we identified *T*_*FCA*_ and *T*_*FCD*_ by identifying the beginning of *T*_*FCA*_ and the end of *T*_*FCD*_ from foot markers and using the fact that |*T*_*FCA*_| ≈ |*T*_*L*_| and |*T*_*FCD*_| ≈ |*T*_*UL*_|.

#### Joint work calculation

To calculate joint moments and velocities and eventually the joint work, we used inverse dynamics using an open source physics engine, DART [22, 23]. We computed the joint moment and velocity from the recorded kinematics, GRF, and CoP. Joint positions *q* were obtained by solving a standard inverse kinematics problem. Next, we derived the joint velocities $\dot{q}$ and accelerations $\ddot{q}$ by taking fourth-order central finite differences of the joint trajectories. Lastly, we obtained the joint moments *τ* from the equations of motion: $M\left(q\right)\ddot{q}+C\left(q,\dot{q}\right)=\tau +J^{T}F$, where *q* is joint positions, *M* is the inertia matrix, *C* is the Coriolis and gravity vector calculated by DART, *J* is a Jacobian matrix and *F* is the GRF. The mass distribution of participants was assumed to follow the adapted inertial parameters in the study by DeLeva [24].

Joint power *P* was calculated by taking the inner product of the instantaneous joint moment and velocity vectors of the leading leg joints during ascent and the trailing leg joints during descent [16]. Positive work in a single DOF during *T*_*L*_ was obtained by taking the integral of *P* when it is positive during *T*_*L*_, denoted by the domain *POS*, such that

$$\begin{array}{c} W_{joint}^{+}=\int_{POS}Pdt, \end{array}$$ (1)

where $W_{joint}^{+}$ is the positive work of a particular joint. Similarly, negative work in a single joint during *T*_*UL*_ was obtained by taking the integral of *P* only when it is negative during *T*_*UL*_, denoted by the domain *NEG*, such that

$$\begin{array}{c} W_{joint}^{-}=\int_{NEG}Pdt, \end{array}$$ (2)

where $W_{joint}^{-}$ is the negative work of a particular joint. To quantify the assistance provided by the ERAS, we obtained individual joint positive work as well as the total positive work generation from all joint DOFs during *T*_*L*_, and individual joint negative work as well as the total negative work generation from all joint DOFs during *T*_*UL*_ (Table 2). Specifically, we calculated the positive work generated by each of the five DOFs of the leading leg (hip-sagittal, hip-frontal, knee, ankle-sagittal, and ankle-frontal joints) during *T*_*L*_. Then, we summed up the positive work generated by these five DOFs to find the total positive work generated by the leading leg during *T*_*L*_ ($W_{TOT}^{+}$). In addition, we summed the positive work generated by the three sagittal DOFs (i.e. by the hip-sagittal, knee and the ankle-sagittal DOFs) to find positive work generation in the sagittal plane only ($W_{Sag}^{+}$). We also calculated the negative work generated by each of the five DOFs of the trailing leg during *T*_*UL*_, the total negative work ($W_{TOT}^{-}$), as well as the negative work generation in the sagittal plane only ($W_{Sag}^{-}$). We repeated these calculations in three conditions; PRE, ASSIST and MATCH.

**Table 2:** Work metrics and their definitions.

Metric	Description	Obtained during
$W_{TOT}^{+}$	Total positive work generated by the leading leg in all DOF	Ascent, *T*_*L*_
$W_{Sag}^{+}$	Total positive work generated by the leading leg in sagittal plane	Ascent, *T*_*L*_
$W_{hip}^{+}$	Positive work generated by the leading-leg hip joint in sagittal plane	Ascent, *T*_*L*_
$W_{knee}^{+}$	Positive work generated by the leading-leg knee joint	Ascent, *T*_*L*_
$W_{ank}^{+}$	Positive work generated by the leading-leg ankle joint in sagittal plane	Ascent, *T*_*L*_
$W_{TOT}^{-}$	Total negative work generated by the trailing leg in all DOF	Descent, *T*_*UL*_
$W_{Sag}^{-}$	Total negative work generated by the trailing leg in sagittal plane	Descent, *T*_*UL*_
$W_{hip}^{-}$	Negative work generated by the trailing-leg hip joint in sagittal plane	Descent, *T*_*UL*_
$W_{knee}^{-}$	Negative work generated by the trailing-leg knee joint	Descent, *T*_*UL*_
$W_{ank}^{-}$	Negative work generated by the trailing-leg ankle joint in sagittal plane	Descent, *T*_*UL*_

#### Statistical analysis

To quantify how much joint work was reduced by using ERAS, we compared the total positive or negative work, the work by all sagittal DOFs, as well as the work by individual DOFs. We compared the work metrics in three conditions (PRE, ASSIST and MATCH) and 9 participants using ANOVA, with both “conditions” and “participants” as fixed factors. Tukey HSD was used to test the mean differences among conditions with significance at *p* < 0.05.

## Results

### Operation of energy-recycling stairs

The ERAS stores energy during the unloading phase (*T*_*UL*_) in stair descent and subsequently returns energy to the user during the loading phase (*T*_*L*_, Figs 1 and 4) in stair ascent. During *T*_*UL*_, CoM lowering begins as the leading leg pushes the movable tread down and stores energy in the extended springs (Fig 4B at ∼70%). Energy storage in the springs is complete when the leading foot (and therefore also the leading movable tread) is fully lowered by the step height (Fig 4B between 88.8% and 100%). As a result, *T*_*UL*_ fully encompasses the time interval during which the storage of energy in the springs would affect negative work during stair descent. During *T*_*L*_, CoM elevation begins with the leading foot contact, which triggers the release of energy from the springs of the lower tread to the trailing leg. Energy release begins shortly after as the trailing foot begins to be elevated (Fig 4A between 0% and 13.6%). Energy release is complete when the trailing foot (and therefore also the trailing movable tread) is fully elevated by the step height (Fig 4A at ∼32%). As a result, *T*_*L*_ fully encompasses the time interval during which the release of energy in the springs affects positive work during gait ascent (See S1 Video).

### Assessment of assistance provided during stair negotiation

All users—ranging from 46 to 100 kg (Table 1)—were successful in stair negotiation on the ERAS. Participants had no prior knowledge about the ERAS, nor were they provided with any information about the purpose of the ERAS during the experiment session. In over 360 trials across 9 participants, no adverse events or concerns about safety were reported.

**Fig 5:**
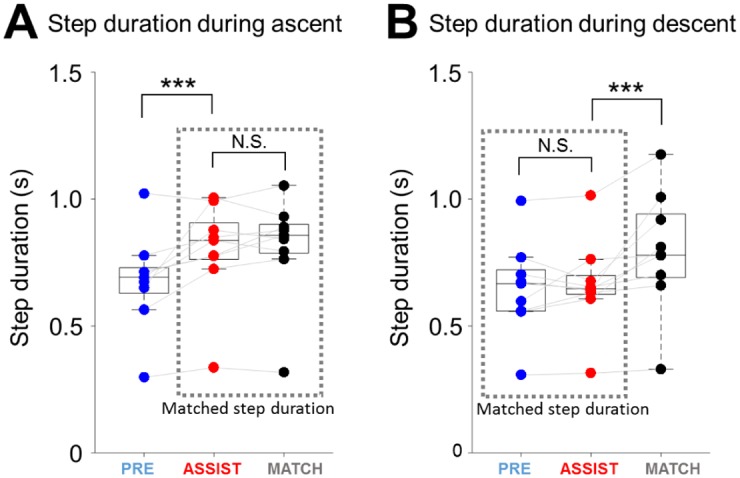
Step duration in different stair negotiation conditions. A) During ascent, step duration on the ERAS was significantly longer than in normal stair negotiation (ASSIST versus PRE). No significant difference was observed in the step duration between ERAS and normal stairs with slower, matched gait speed (ASSIST versus MATCH). Thus, we compared the results (ASSIST) against MATCH (instead of PRE) during ascent. B) During descent, step duration was not different on ERAS versus during normal stair negotiation (ASSIST versus PRE). However, the descent steps in the MATCH condition were significantly longer than on ERAS (ASSIST versus MATCH). Thus, we compared the results (ASSIST) against PRE (instead of MATCH). *** refers to *p* < 0.001, * refers to *p* < 0.05, and N.S. refers to no significant difference.

During ascent, the step duration in the ASSIST condition was significantly longer than the step duration in the PRE condition (ASSIST versus PRE, *p* < 0.001), but was not different from the step duration in the MATCH condition (ASSIST versus MATCH, *p* > 0.5, Fig 5A). During descent, the step duration during the ASSIST condition was not different from the step duration in the PRE condition (ASSIST versus PRE, *p* > 0.5), but was significantly shorter than the step duration in the MATCH condition (MATCH versus ASSIST, *p* < 0.001, Fig 5B). In other words, in the ASSIST condition, stair ascent was slower than during normal stair negotiation (PRE) but descent was at a comparable speed. This counter-intuitive observation is discussed later in Discussion. Based on step duration, we used the MATCH condition as our speed-matched control for the ASSIST condition during ascent, and used the PRE condition as our speed-matched control for the ASSIST condition during descent.

**Fig 6:**
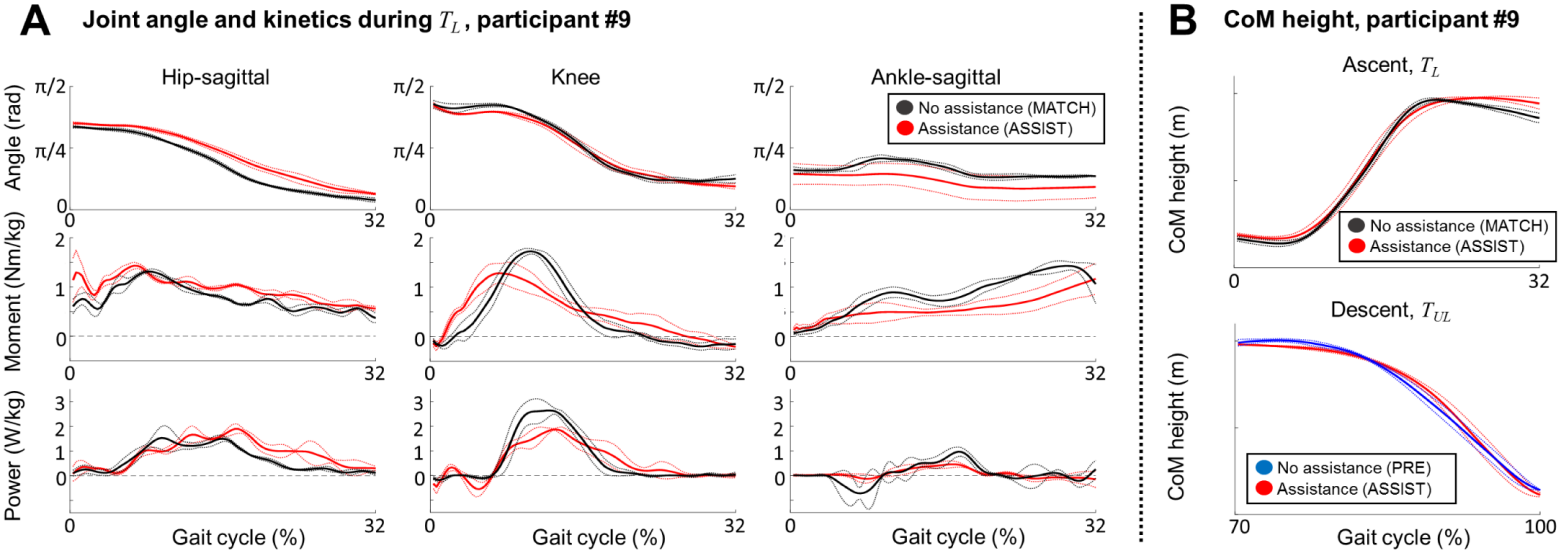
Joint kinematics and center-of-mass trajectories of participant #9. A) Joint angle, moment, and power for the hip-sagittal, knee and ankle-sagittal DOFs during *T*_*L*_ (0%-32% of the gait cycle during ascent) for ASSIST (red) and MATCH (black) conditions. The solid line indicates the mean trajectory and the dashed lines indicate one standard deviation. B) Center-of-mass height over time during *T*_*L*_ (top), and during *T*_*UL*_ (bottom). Blue trajectories indicate PRE condition.

ERAS did not qualitatively affect movement kinematics or CoM motion, consistent with self-reports of the ERAS being easy to use. Fig 6 shows the joint angle and CoM trajectories of a representative participant (#9). Sagittal plane joint angles of the hip, knee, and ankle were qualitatively similar during *T*_*L*_ between the ASSIST and the MATCH conditions, with the hip-sagittal angle slightly higher and the ankle-sagittal angle slightly lower than in MATCH. The CoM height from the ground also followed similar trajectories between ASSIST and MATCH conditions (ascent) as well as between ASSIST and PRE conditions (descent). There was no evidence of sudden lifts or drops of CoM.

#### Stair ascent

**Fig 7:**
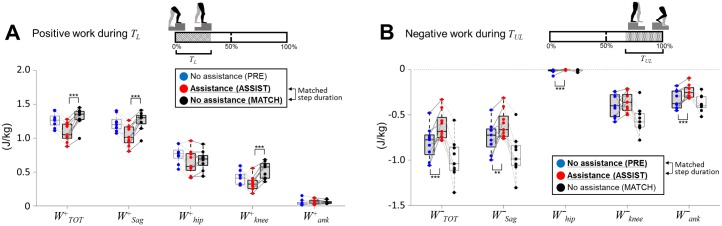
Positive and negative work on ERAS. Work metrics in the ASSIST condition (shaded boxes, red dots) and in speed-matched control conditions (MATCH for ascent, PRE for descent). Thin and dark gray lines connect each participant’s result in blue, red, and black dots. A) Ascent: Between ASSIST and MATCH conditions, $W_{TOT}^{+}$, $W_{Sag}^{+}$ and $W_{knee}^{+}$ were reduced by 17.4 ± 6.9%, 17.8 ± 7.3% and 37.7 ± 10.5%, respectively. PRE trials are also shown for reference (white boxes, blue dots). B) Descent: Between ASSIST and PRE conditions, $W_{TOT}^{-}$, $W_{Sag}^{-}$ and $W_{ank}^{-}$ were reduced by 21.9 ± 17.8%, 16.9 ± 21.3% and 26.0 ± 15.9%, respectively. $W_{hip}^{-}$ was also significantly reduced, but the absolute reduction is very small. MATCH trials are also shown for reference (white boxes, black dots). Significance of *p* < 0.001 are noted as ***, *p* < 0.01 are noted as **.

In speed-matched normal stair ascent (MATCH), the total positive work of 1.317 ± 0.134 J/kg was generated by the leading leg during *T*_*L*_ ($W_{TOT}^{+}$, Fig 7A). The sagittal-plane DOFs (hip-sagittal, knee and ankle-sagittal) generated over 95.8 ± 2.1% ($W_{Sag}^{+}$, 1.262 ± 0.137 J/kg) of the total positive work. The hip-sagittal DOF generated 51.4 ± 8.1% ($W_{hip}^{+}$, 0.677 ± 0.107 J/kg), the knee DOF generated 40.0 ± 7.9% ($W_{knee}^{+}$, 0.527 ± 0.104 J/kg), and the ankle-sagittal DOF generated 4.2 ± 2.7% ($W_{ank}^{+}$, 0.056 ± 0.036 J/kg) of the total positive work during *T*_*L*_. Contribution of frontal DOFs (hip-frontal and ankle frontal) to total positive work was 4.4 ± 2.0%.

Using ERAS significantly reduced positive work generation during *T*_*L*_. When speed was matched, using ERAS reduced $W_{TOT}^{+}$ by 17.4 ± 6.9% (ASSIST versus MATCH, *p* < 0.001, Fig 7A). $W_{knee}^{+}$ was reduced by 37.7 ± 10.5% (ASSIST versus MATCH, *p* < 0.001). However, $W_{hip}^{+}$ did not change significantly (ASSIST versus MATCH, *p* > 0.25).

**Fig 8:**
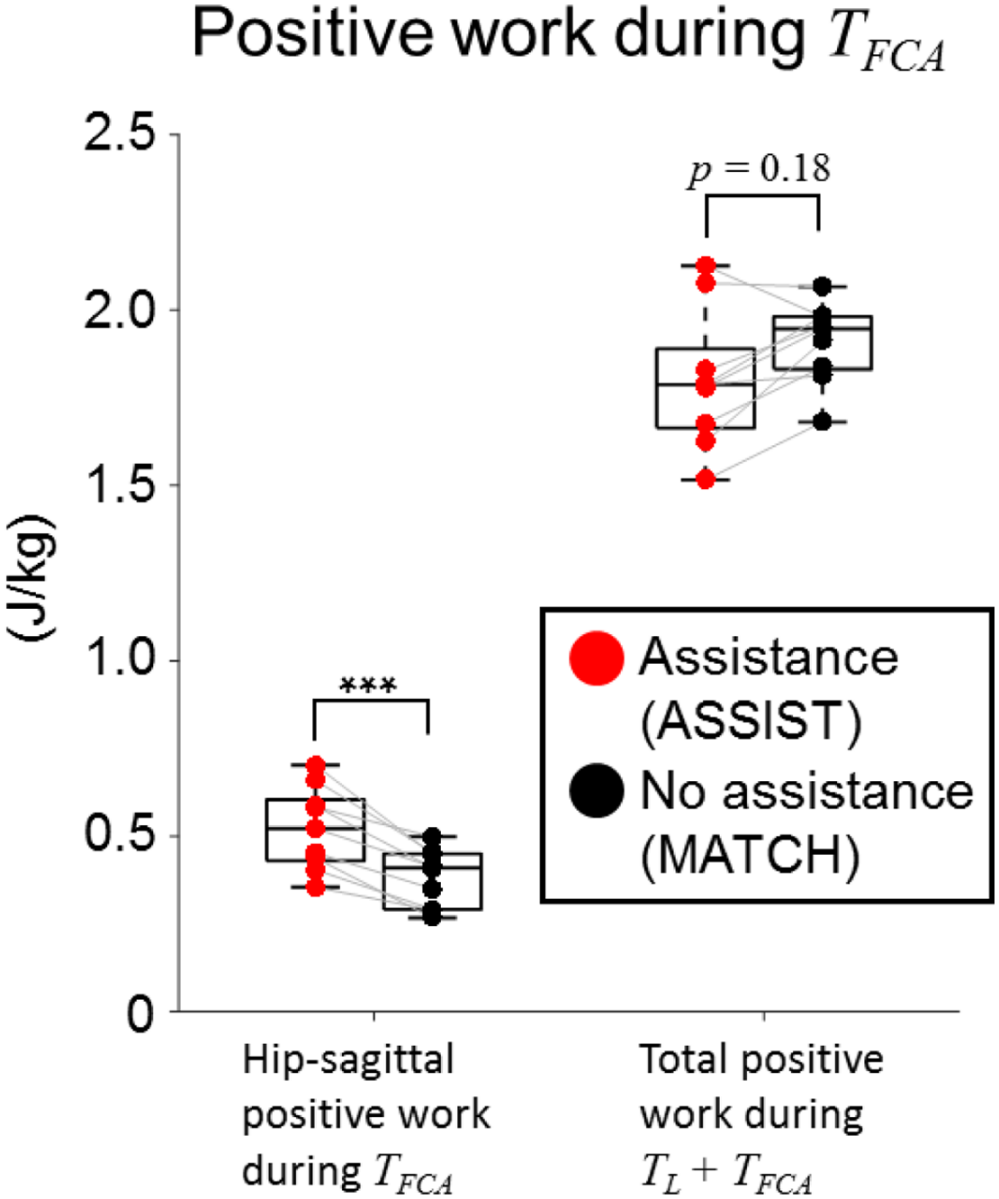
Positive work generated during *T*_*FCA*_. Positive work generated by the hip-sagittal DOF was significantly higher in the ASSIST condition (0.523 ± 0.119 J/kg) versus the MATCH condition (0.381 ± 0.084 J/kg, *p* < 0.001). The total positive work generated during *T*_*L*_ + *T*_*FCA*_ was not significantly different, with a trend towards slight reduction in ASSIST from MATCH (0.088 ± 0.101 J/kg, *p* < 0.2).

During the forward-continuance phase of ascent, *T*_*FCA*_, positive joint work generated by each DOF showed no difference between ASSIST and MATCH conditions, except for the hip-sagittal DOF, which generated 0.142 ± 0.063 J/kg more positive work during ASSIST than in MATCH (Fig 8). However, because the total positive work reduction during *T*_*L*_ was 0.230 ± 0.094 J/kg, the total positive work during *T*_*L*_ + *T*_*FCA*_ = [0%, 64%] showed a trend of reduction by 0.088 ± 0.108 J/kg (*p* < 0.2).

#### Stair descent

In speed-matched normal stair ascent (PRE), the total negative work of −0.807 ± 0.179 J/kg was generated by the leading leg during *T*_*UL*_ ($W_{TOT}^{-}$, Fig 7B). The sagittal-plane DOFs (hip-sagittal, knee and ankle-sagittal) generated 92.2 ± 4.2% ($W_{Sag}^{-}$, −0.744 ± 0.169 J/kg) of the total negative work. The knee DOF generated 48.7 ± 18.8% ($W_{knee}^{-}$, −0.393 ± 0.152 J/kg), the ankle-sagittal DOF generated 41.5 ± 15.3% ($W_{ank}^{-}$, −0.335 ± 0.123 J/kg), and the hip-sagittal DOF generated 1.9 ± 3.4% ($W_{hip}^{-}$, −0.016 ± 0.028 J/kg) of the total negative work during *T*_*UL*_. Contribution of frontal DOFs (hip-frontal and ankle-frontal) to total negative work was 7.9 ± 5.0%.

Using ERAS significantly reduced negative work generation during *T*_*UL*_. When speed was matched, using ERAS reduced $W_{TOT}^{-}$ by 21.9 ± 17.8% (PRE versus ASSIST, *p* < 0.001, Fig 7B). $W_{ank}^{-}$ was reduced by 26.0 ± 15.9% (PRE versus ASSIST, *p* < 0.001). However, $W_{knee}^{-}$ did not change significantly (PRE versus ASSIST, *p* > 0.68).

Using ERAS did not change the total joint work during the forward-continuance phase during descent, *T*_*FCD*_. Negative work generated by each DOFs, as well as the total of them, were not different between PRE and ASSIST conditions.

## Discussion

Our energy-recycling assistive stairs recycle energy from human movement to assist stair negotiation without the use of high-power actuators. Naive users with no prior exposure to ERAS were able to safely and effectively use our stairs, which operates with a system of springs and movable treads. We show that ERAS stores energy that is usually absorbed by the trailing leg during stair descent. Although further investigation is required, this reduction in negative work could possibly reduce the metabolic cost of stair descent, and also reduce muscle and joint forces that cause pain [25, 26]. Similarly, stored energy in the ERAS is returned to users during stair ascent, showing a trend towards reducing the amount of positive work generated by the user. This could also lead to reduced metabolic energy as well as muscle and joint forces during stair ascent.

### Implications of ERAS as an assistive device

ERAS is the first non-wearable device that recycles human-generated mechanical energy to provide motor assistance (Table 3). Several existing wearable energy-recycling devices convert mechanical energy stored from human movement into electrical energy to power external devices [14, 27–35]. However, converting mechanical energy to electrical energy is inefficient and unnecessary for applications requiring mechanical energy output for movement assistance. Wearable devices [14, 15, 28–33] are constrained in the amount of energy that can be stored and released due to tight constraints on space and weight. By installing the energy recycling mechanism on a staircase, the ERAS can use large (and possibly heavier) springs which allow for a greater amount of energy storage, without the form factor constraints imposed by wearable devices. As user compliance in wearing assistive devices can be limited, modifications in the home environment may be more beneficial in providing greater independence, safety, and mobility to those with difficulty with stair negotiation. Moreover, while all other existing energy-recycling devices available target overground human locomotion such as walking or running, ERAS is the only device tailored for assistance during stair negotiation.

**Table 3:** Current energy-recycling devices.

	Non-wearable	Wearable
Output: mechanical energy for human movement assistance	ERAS (this work)	Collins 2015 [15]
Output: electrical energy for external device operations	Pavegen^®^ [27]	Niu 2004 [28], Hayashida 2000 [29], Paradiso 2005 [30], Riemer 2010 [31], Donelan 2008 [14], Rome 2005 [32], Granstrom 2007 [33]

Energy-recycling devices in the literature are categorized based on their configuration (wearable versus non-wearable) and energy output (mechanical versus electrical energy). The underlined device is for stair negotiation; all other devices are for level walking.

For people with reduced motor function who can still negotiate stairs with some assistance, our modular and low-power ERAS is a low-cost and effective alternative to the existing high-power and expensive options such as elevators, escalators or stair lifts. The modular design of ERAS allows quick and easy installation (and removal, if necessary) on top of an existing staircase without expensive remodeling. The passive springs, low-power electromagnetic latches, and a single Arduino board together require very little power compared to motorized devices, thereby reducing the cost of use. The low-cost and low-power design allows ERAS to be more affordable and practical to people with limited financial resources. The modular ERAS can easily integrate onto existing stairs, making homes and communities more suited for aging-in-place [36].

One surprising outcome of ERAS is its ability to assist not only stair ascent, but also stair descent. Perhaps counter-intuitively, stair descent can be as energy-demanding as stair ascent. A person (body weight = *mg*) ascending a step (height = *h*) must generate net positive energy in the legs of *mgh*. Similarly, descending a step requires a potential energy of *mgh* to be dissipated by negative work generation in the legs and through collision. Hence, if energy lost through collision is small, the negative work during descent can be of similar magnitude to positive work in ascent. In fact, Fig 7 show that $W_{TOT}^{-}$ is comparable in magnitude to $W_{TOT}^{+}$. Moreover, the control of muscle braking is more challenging than generating positive work with muscles, and is a focus of exercises to help frail older adults to better descend stairs and to decrease the risk of fall [37].

While the current hardware prototype allows initial investigation of the assistance provided by ERAS, future evolutions of ERAS could allow higher usability in practical settings. To use the current prototype, a user needs to first descend and then ascend the ERAS modules in order to store and then to return mechanical work. This constraint can be eliminated in the future prototypes by incorporating additional mechanisms to configure the ERAS to either “ascend-able” (treads down) or “descend-able” (treads up) positions, thereby also allowing multiple users to consecutively ascend or descend the ERAS modules.

### Experimental limitations and justifications

The mis-match in the descent step duration between ASSIST and MATCH conditions (Fig 5) may be due to the verbal instructions to the participants as well as the tempo of the audio beat. We informed the participants that they might walk more slowly in the following trials than they would normally on normal stairs. This may have caused some participants to be predisposed to slow down during both ascent and descent compared to PRE trials. However, since stair descent was not slower in ASSIST than in PRE, stair descent in MATCH turned out to be slower than in ASSIST. Also, the audio beat we selected was similar to the speed of ascent of the last ASSIST trial, but was too slow for the descent. Speed-match trials in future experiments should be conducted with refined instructions and separate audio beat tempos for ascent and descent to more accurately represent the step durations during ASSIST.

We only examined joint work as a first step, but further study on the specific effects of ERAS on more physiologically-relevant metrics is warranted, particularly in mobility-limited individuals. These could include joint range of motion [6], peak joint moment/power [1–3], intra-articular joint forces [38], muscle co-activation [39, 40], net metabolic energy reduction [15] or the amount of eccentric muscle contraction required [41]. As a first step, we assessed the efficacy of the ERAS in providing assistance using measures of joint work. Although our metrics demonstrate that mechanical work was stored and returned to the user, it is still possible that participants co-activated muscles to stiffen the joints when using the ERAS, which could increase metabolic energy, muscle forces, and inter-joint forces; it is not clear whether such changes would increase or reduce joint pain. Factors specific to mobility limitations in specific populations also need to be directly studied when considering the appropriateness of ERAS in providing assistance in stair negotiation.

We were unable to measure the reaction forces on the movable stair treads, but believe that measures from the top landing are comparable to those from intermediate steps on the ERAS. Gait patterns are similar in stair ascent when stepping on the top landing as when stepping onto the intermediate ERAS steps with the tread in the lowered position. Similarly, in stair descent lowering the body from the top landing is similar to lowering the body on the ERAS with the tread locked in the lowered position. Although the ideal case would be to measure GRFs on the moving treads, this would require very light and thin force plates, or for the ERAS modules to be isolated and mounted on stationary force plates [2].

### Summary

Our promising results that show energy-recycling during stair negotiation in young healthy participants motivate further refinement and optimization of the Energy-Recycling Assistive Stairs to aid older adults and individuals with a wide range of mobility impairments. As healthy users could safely benefit from ERAS without explicit instructions or training, more effective user guidelines and practice could facilitate stair negotiation for those with muscle weakness or joint pain. The physical design of the ERAS could be tailored to provide more user-specific trajectories of energy storage and release. Further reduction in the net electrical energy expenditure could also be achieved through a more refined mechanical design and well as the harvesting energy from stair motion to power the system’s electronics. It is possible that each ERAS could be operated independently without external power. In addition, future ERAS could overcome the limitations of the current prototype, such as to provide assistance to multiple users. Overall, our proof-of-principle demonstration provides a novel platform for interactive, personalized, energy-efficient, and cost-effective devices for assisting stair negotiation to suit a wide range of individuals with reduced mobility.

## Supporting information

S1 Video ERAS operation demonstration. A user descending and ascending a staircase with two ERAS modules.
(MP4)
